# Selective expression of *Pneumocystis* antigens in different patients during a suspected outbreak of *Pneumocystis* pneumonia

**DOI:** 10.1128/mbio.00692-25

**Published:** 2025-04-17

**Authors:** Caroline S. Meier, Marco Pagni, Sophie Richard, Konrad Mühlethaler, Philippe M. Hauser

**Affiliations:** 1 Institute of Microbiology, Lausanne University Hospital and University of Lausanne 536517, Lausanne, Switzerland; 2 Vital-IT Group, SIB Swiss Institute of Bioinformatics 30489, Lausanne, Switzerland; 3 Institute for Infectious Diseases, University of Bern 87618, Bern, Switzerland; Albert Einstein College of Medicine, Bronx, New York, USA

**Keywords:** *Pneumocystis jirovecii*, *Pneumocystis *pneumonia, PCP, PJP, cluster, major surface glycoprotein, genotyping, PacBio circular consensus sequencing

## Abstract

The fungus *Pneumocystis jirovecii* causes severe pneumonia in immunocompromised individuals. It harbors a system of surface antigenic variation involving six families of major surface glycoproteins (Msg). We determined the repertoires of *P. jirovecii msg* genes of the most abundant family-I present in 15 Swiss patients with *Pneumocystis* pneumonia (PCP) enrolled randomly. The highly repetitive *msg*-I genes were sequenced using generic PCRs followed by circular consensus sequencing with long reads. In contrast to the other 12 patients, three renal transplant recipients (RTRs) harbored the same repertoire of *msg*-I genes. Multilocus genotyping showed that these RTRs were infected by the same *P. jirovecii* genotype that differed from those present in the other 12 patients. These observations suggested that these RTRs were involved in an outbreak of PCP due to interhuman transmission or a common source of the fungus. Although they harbored the same repertoire of *msg*-I genes, the sets of *msg*-I genes that were expressed differed between the three patients. This suggested that selective expression of *P. jirovecii* surface antigens might have played a role in the pathogenesis of PCP by allowing escape from the immune response specific to each patient. Although expected for a family of genes, this is the first time that selective expression of antigens is observed in *Pneumocystis*. The previously described adaptation of *P. jirovecii* to infect solid organ transplant (SOT) recipients through resistance to the immunosuppressant mycophenolate probably also favored the suspected outbreak. Moreover, our study supports the idea that various *P. jirovecii* genotypes can adapt to infect SOT recipients.

**IMPORTANCE:**

The fungus *Pneumocystis* causes severe pneumonia in patients with weakened immune systems. It possesses a genetic system to vary the antigens at the surface of its cells that are presented to the immune system of the patient. We report for the first time that this system may have been implicated in the infections of renal transplant recipients involved in a suspected outbreak. Our observations suggest that the antigens presented might be selected to avoid the elimination of the fungus by the immune response specific to each patient. The resistance of the fungus to the immunosuppressant mycophenolate administered to these patients to prevent organ rejection probably also played a role in the infections during the suspected outbreak.

## INTRODUCTION

The opportunistic fungus *Pneumocystis jirovecii* colonizes human lungs and causes severe pneumonia in immunocompromised individuals (1). These individuals include patients infected by HIV, cancer, inflammatory or autoimmune diseases, as well as transplant recipients of solid organs or stem cells. Over the past few decades, *Pneumocystis* pneumonia (PCP) has been one of the most frequent fungal invasive infections with approximately half a million cases reported annually worldwide (2). Despite its prevalence, no method of long-term *in vitro* culture has been established for this pathogen. Two methods have been proposed (3, 4), but none have been reproduced so far (5).

PCP was initially believed to result from the reactivation of the fungus acquired during an infantile primary infection. However, the transmission of *Pneumocystis* from host to host by airborne particles has been demonstrated by molecular analysis of outbreaks of PCPs among solid organ transplant (SOT) recipients (6–9) as well as experiments in animal models (10, 11). These airborne particles are the asci and/or the ascospores produced during the sexual cycle of the fungus (10–12). Currently, the primary reservoir of the fungus is believed to consist of individuals who are colonized (13, 14).

The finding of the same *P. jirovecii* genotype in SOT recipients involved in three outbreaks of PCP that occurred ca. 200 km apart in Europe suggested a specific adaptation of the fungus to infect these patients (7). Different genotypes were observed in other such outbreaks indicating that adapted genotypes may emerge independently (15). In France, a genotype adapted to SOT recipients harbored a mutation in the inosine monophosphate dehydrogenase (IMPDH) conferring potential resistance to mycophenolate (16), an immunosuppressant widely used in these patients. Mycophenolate and similar drugs also target enzymes of opportunistic organisms, including *Pneumocystis*. Besides, mutations in the *P. jirovecii* dihydropteroate synthase (DHPS) which may confer resistance to sulfonamides likely contributed to the failure of PCP prophylaxis in another outbreak (17).

Additional factors may be involved in the development of infection upon interhuman transmission of *Pneumocystis* during outbreaks. Immune responses may vary depending on the *Pneumocystis* antigens previously encountered and the characteristics of the patient. Selective pressure from the immune system likely acts on the major surface glycoproteins (Msg), which cover all *P. jirovecii* microorganisms (18–21). Presumably, to evade this pressure, *P. jirovecii* possesses a system for surface antigenic variation. Our model (22) proposes that this system relies on gene mosaicism of six families of Msg and the reassortment of the approximately 80 subtelomeric *msg* alleles of family-I, the most abundant Msg (19, 20, 22). Over time, single *msg*-I alleles are retrieved from this repertoire for mutually exclusive expression by translocation downstream of a promoter present at a single copy per genome. This mechanism would generate subpopulations of microorganisms expressing different antigens that accumulate during the growth of the fungus. Consistent with the hypothesis that this system allows evading the human defense mechanisms, Msg is immunogenic (1), and variation of the expressed *msg* alleles was described in the rat model of *Pneumocystis* infection (23).

Among 15 patients with PCP enrolled randomly in two Swiss cities, we observed a specific repertoire of *P. jirovecii msg*-I alleles in each patient, except in three renal transplant recipients (RTRs) who harbored the same one. This suggested that the three RTRs were involved in an outbreak of PCP. The first objective of the present study was to assess the suspected outbreak by determining if, as expected for an outbreak, the three RTRs were infected by the same single *P. jirovecii* genotype. The second objective was to take advantage of the presence of the same repertoire of *msg*-I alleles in different patients to investigate the potential role of the antigenic variation of *P. jirovecii* in the pathogenesis of PCP. To that second objective, we determined the *msg*-I alleles that were expressed in the patients.

## RESULTS

### Suspicion of an outbreak of PCP among RTRs by the analysis of the repertoires of *P. jirovecii msg*-I genes

**Fig 1:**
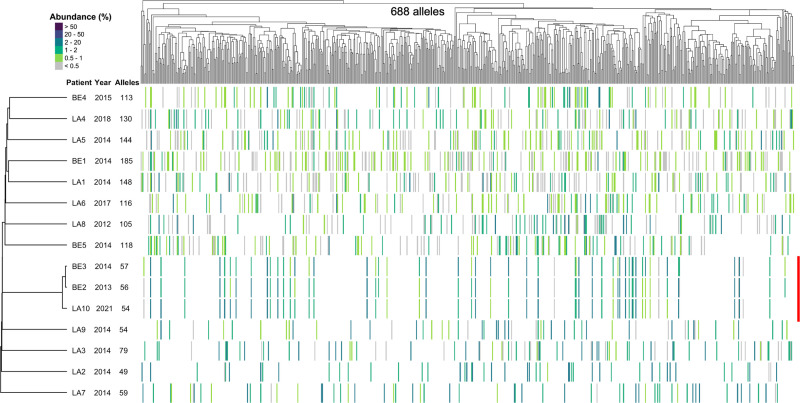
Composition of the *msg*-I genomic repertoires present in 15 Swiss patients with PCP, including three RTRs involved in a suspected outbreak. The vertical red line on the right identifies the suspected outbreak. The collection year and the number of alleles present in the patient are indicated next to the patient’s code. Each vertical line of the heatmap represents an allele present in the given repertoire, with the color representing its abundance in the percentage of all reads composing the repertoire, as indicated at the top left of the figure. The 688 distinct alleles identified in the repertoires were sorted using a hierarchical classification tree of the multiple alignments of the allele sequences (Fitch distance, average linkage). The patients were sorted using a tree of the presence/absence of each allele in their repertoire (binary distance, average linkage). The *msg*-I repertoires shown in this figure, except those of patients BE3 and LA10, were previously published using classification trees including more alleles present in more patients (**Fig. 1**[22]). LA, Lausanne; BE, Bern. Data are provided in Table S2.

We determined the “genomic” repertoires of *P. jirovecii msg*-I alleles present in 15 Swiss immunocompromised patients with PCP, enrolled randomly, 10 in the city of Lausanne and five in the city of Bern. There were six HIV-infected patients, three RTRs, two patients with cancer, and four with unknown causes of immunosuppression (Table S1). The “genomic” repertoires correspond to the approximately 80 non-expressed *msg*-I alleles per genome plus those that are expressed in each *P. jirovecii* population. To characterize the repertoires of these highly repetitive genes, we used a previously developed procedure using PacBio circular consensus sequencing (CCS) of generic PCR products with long reads, followed by bioinformatics to identify and quantify the alleles (22). Twelve of the 15 patients harbored distinct genomic repertoires including 49–185 alleles (Fig. 1). The repertoires with more than 80 alleles result from the frequent co-infections with several *P. jirovecii* genotypes harboring different repertoires, while those with less than 80 results from the limitation of the method to detect some alleles (see Supplementary Information in Supplemental Material). The remaining three patients BE2, BE3, and LA10 harbored almost identical repertoires made of respectively 56, 57, and 54 alleles (emphasized by a red line on the right of Fig. 1). These three patients were RTRs and the only SOT recipients among the 15 patients analyzed. Two of them were diagnosed in Bern 7 months apart (BE2 and BE3) and the third in Lausanne 7 years later (LA10). The presence of almost the same genomic repertoire of *msg*-I alleles and the same underlying disease in the three RTRs suggested the possibility of an outbreak of PCP caused by a single *P. jirovecii* genotype adapted to infect these patients.

### Single *P. jirovecii* genotype resistant to mycophenolate in the suspected outbreak

The hypothesis of an outbreak was further investigated by multilocus sequence typing of *P. jirovecii* present in the patients. Indeed, a hallmark of the outbreaks among SOT recipients is the presence of the same single *P. jirovecii* genotype in all patients involved (6–9, 17). We also addressed the presence of the mutations in the IMPDH and DHPS genes that potentially confer resistance to mycophenolate and sulfonamides, respectively, as these were observed previously in SOT recipients (16, 17). All patients not involved in the presumed outbreak were co-infected with several genotypes, except patient LA2 (patients “Swiss non-outbreak,” Table 1; co-infections are revealed by the presence of several alleles of at least one locus as *P. jirovecii* is haploid). The genotypes in these non-outbreak patients were diverse and did not harbor resistance mutations, except those infecting patient LA3 with a mutated DHPS. In contrast, the three RTRs of the suspected outbreak were infected by the same single *P. jirovecii* genotype differing from those present in the other 12 patients and that carried a resistance mutation in IMPDH, but not in DHPS (patients “Swiss suspected outbreak,” Table 1; patient LA10 harbored also a second minority genotype evidenced by sequencing the mt26S locus). To further investigate the issue, we genotyped *P. jirovecii* present in four supplementary RTRs from Bern that were contemporary to the suspected outbreak. Three of them were infected with the same single resistant genotype and were consequently part of the suspected outbreak. The fourth RTR harbored at least two other non-resistant genotypes (“Bern RTR transplant recipients,” Table 1). The finding of the same single genotype in six RTRs that differed from those present in the other patients was consistent with an outbreak of PCP among SOT recipients.

**TABLE 1:** Multilocus sequence genotyping of *P. jirovecii* from patients with PCP^*a*^

Patient code/genotype	Number of genotypes	ITS1	26S	mt26S	b-tub	CytB	DHPS^*b*^	IMPDH^*c*^
Swiss non-outbreak								
LA1	>2	B (9), B2 (3), B3 (1), and B7 (1)	1	7 (11)	1 and 3	Mix^*d*^	Wild type	Wild type
LA2	1	B (13)	1 (14)	8 (16)	1 (15)	1	Wild type	Wild type
LA3	2	B (12)	1 (1) and 4 (4)	8 (5)	3 (5)	2	M2	Wild type
LA4	≥2	B3 (8)	1 (4) and 9 (4)	8	1 and 3	2	Wild type	Wild type
LA5	>2	A1 (3), B (11), and B7 (1)	2 (6)	7 (11) and 8 (3)	1 (2) and 3 (10)	5 and 8	Wild type	Wild type
LA6	≥2	B (8) and B2 (4)	1 (5) and 9 (3)	2 and 8	1 and 3	1 and 7	Wild type	Wild type
LA7	2	A2 (5)	4 (6)	1 (1) and 2 (4)	3 (5)	2	Wild type	Wild type
LA8	≥2	B (19)	4 (1) and 6 (4)	2 (2), 3 (2), 8 (3), and 15 (1)	3 (9)	2	Wild type	Wild type
LA9	2	B (19) and B2 (5)	1	2	1	1	Wild type	Wild type
BE1	>2	A2 (2), A3 (7), and B (3)	6 (4)	2 (1) and 3 (4)	1 and 3	1 and 2	Wild type	Wild type
BE4	≥2	A2 (9) and B (1)	1 (4)	3 (5)	3	4	Wild type	Wild type
BE5	>2	B (7), B3 (1), and B8 (1)	1 (4)	7 (3) and 8 (2)	1 and 3	Mix	Wild type	Wild type
Swiss-suspected outbreak (2013–2021)								
LA10	2	B (15)	1 (15)	2 (1) and 3 (14)	1 (15)	2 (5)	Wild type	M
BE2	1	B (13)	1 (13)	3 (14)	1 (13)	2 (5)	Wild type	M
BE3	1	B (8)	1 (5)	3 (5)	1 (5)	2 (5)	Wild type	M
Bern RTR recipients (2013–2016)								
BE6	1	B	1	3	1	2 (5)	Wild type	M
BE7	1	B (9)	1	3	1	2 (5)	Wild type	M
BE8	1	B	1	3	1	2	Wild type	M
BE9	2	A2 (3) and A3 (8)	1	8	1	2	Wild type	Wild type
Lyon outbreak (1994–1996)								
RTR 1	1	B	1	8	1	4	M2	Wild type
RTR 2	1	B	1	8	1	4	M2	Wild type
HIV	1	B	1	8	1	4	M2	Wild type
Danish transplant recipient outbreaks (2007–2010)								
Genotype outbreak 1	–^*e*^	A1	1	3	–	–	–	–
Genotype outbreak 2	–	B	11	3	–	–	–	–
Genotype outbreak 3	–	B2	1	8	–	–	–	–
Swiss-German outbreaks (2005–2008)								
Genotype	–	B	5	7	1	–	–	–

^*a*^ Multilocus genotyping was performed by sequencing plasmid subclones from each PCR product or direct sequencing of the PCR product. The parentheses show the number of subclones with the sequence of the given allele, the absence of parentheses corresponds to direct sequencing. The presence of several alleles of at least one marker reveals a co-infection with more than one *P. jirovecii* genotype. ITS1, internal transcribed spacer number 1 of the nuclear rRNA genes operon. 26S, intron of the nuclear 26S rRNA gene. mt26S, variable region of the mitochondrial 26S rRNA gene. β-tub, the region surrounding intron number 6 of the β-tubulin gene. CytB, internal half of cytochrome b. DHPS, the region spanning the mutation site in DHPS. IMPDH, the region spanning the mutation site in the inosine 5′-monophosphate dehydrogenase. See “Materials and Methods” for allele naming and numbering.

^*b*^ “Wild type” and mutated “M2” DHPS alleles were obtained by direct sequencing. Their presence in the co-infected patients was assessed by inspection of the sequence chromatogram at the mutations’ positions, excluding a subpopulation with the other allele representing more than approximately 15%. No mixed cases were observed. The mutated M2 allele corresponds to T at position 171 instead of C (24).

^*c*^ “Wild type” and mutated “M” IMPDH alleles were also obtained by direct sequencing and chromatogram assessment for co-infected patients. No mixed cases were observed. The mutated M allele corresponds to A instead of G at position 1,020 in reference 16. This A was observed only in the allele no. 3 in reference 16, whereas the G is present in several other reported alleles that we could not distinguish by the partial sequencing of the locus that we used here.

^*d*^ Direct sequencing did not allow identification of the alleles present in the mix.

^*e*^ "-" indicates not relevant or allele not available.

### Comparison of the genotypes involved in outbreaks among SOT recipients

To better understand the etiology of the outbreaks among SOT recipients, we compared the genotype we observed here to those involved in other such outbreaks previously reported. We genotyped *P. jirovecii* present in two RTRs and one HIV-positive patient involved in an outbreak that we reported in Lyon, France, 21 years ago (17). The genotype was different and carried a resistance mutation in DHPS but not in IMPDH (“Lyon outbreak,” Table 1). We also compared the genotypes reported in four earlier outbreaks that occurred in Europe among RTRs and liver transplant recipients. All four were different, as revealed by the presence of a different allele of at least one marker (“Danish transplant recipient outbreaks” [8] and “Swiss-German outbreaks” [7], Table 1). These observations suggested that various *P. jirovecii* genotypes can adapt to infect SOT recipients.

### Different *P. jirovecii msg*-I expressed repertoires in the RTRs of the suspected outbreak

**Fig 2:**
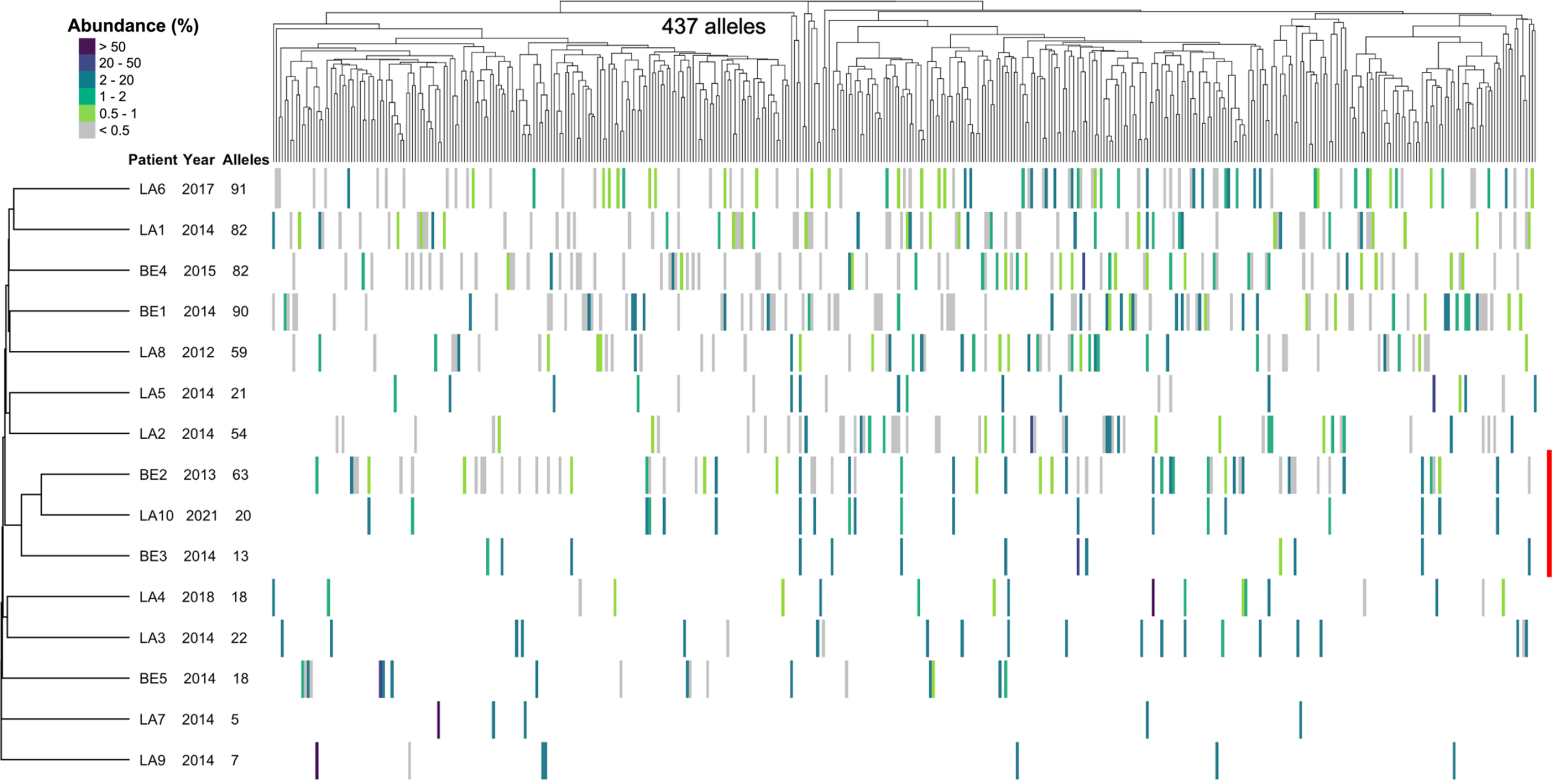
Composition of the *msg*-I expressed repertoires present in 15 Swiss patients with PCP, including three RTRs involved in a suspected outbreak. For legend, see Fig. 1.

Our second objective was to take advantage of the presence of almost the same repertoire in different patients to investigate the potential role of the antigenic variation of *P. jirovecii* in the pathogenesis of PCP. To that aim, we determined the “expressed” repertoires of *P. jirovecii msg*-I alleles present in the 15 patients using a generic PCR that amplifies specifically these alleles (22). All patients harbored distinct expressed repertoires consisting of 5–91 alleles, including the three RTRs of the suspected outbreak (emphasized by a red line on the right of Fig. 2). Nevertheless, these three patients harbored related expressed repertoires as evidenced by the tree on the left of Fig. 2. Indeed, the expressed repertoires present in patients BE3 and LA10 were different subsets of their shared genomic repertoire, whereas alleles of this repertoire were expressed in patient B2 (13, 20, and 63 alleles). However, the composition of the expressed repertoires determined using our approach presents a reduced accuracy and an increased underestimation of the diversity relative to the genomic repertoires (see Supplementary Information in Supplemental Material). Consequently, we further assessed these three expressed repertoires by repeating their analysis twice (Fig. S1; Table S3). Despite significant variation among those of BE3, the triplicates of each expressed repertoire formed a separate branch of the tree sorting the patients, confirming their differences. These observations suggest that selective expression of the *msg*-I alleles from almost the same genomic repertoires occurred in patients from the suspected outbreak.

## DISCUSSION

Among 15 patients with PCP enrolled randomly, we suspect that three RTRs were involved in an outbreak of PCP because they were infected by the same single *P. jirovecii* genotype that differed from those present in the other 12 patients analyzed. The presence of the same repertoire of *msg*-I alleles in these three different patients allowed the investigation of the potential role of the antigenic variation of *P. jirovecii* in the pathogenesis of PCP. Our goal has not been to formally demonstrate the outbreak by investigating possible interhuman transmission or a common source of the fungus, as already done in numerous studies (6–9, 17). The lack of this demonstration does not impact the conclusions of our study because they are not based on the reasons for the presence of the same genomic repertoire in different patients.

The suspected outbreak would have lasted at least 8 years because the six RTRs involved were diagnosed over this period, from 2013 to 2021. Such duration is like previous observations in outbreaks among SOT recipients (7, 8, 17). There is a maximum gap of 5 years between the diagnoses of two patients among the six patients of the suspected outbreak (patients B6 and LA10). It is not excluded that *P. jirovecii* has been transmitted directly between these two patients because social meetings are frequent among SOT recipients, and Switzerland is a small country. Nevertheless, intermediary hosts may have been involved, such as unknown SOT recipients or colonized immunocompetent individuals. The detection of *P. jirovecii* DNA in the dust in the homes of patients with PCP (25) suggests the further possibility of infections by a form of the fungus dormant in the environment. Such a state has been previously envisaged to account for discrepant results concerning the structure of *P. jirovecii* populations (26). Despite the presence of the resistant genotype in the other RTRs of Bern, one RTR of this city contemporary to the suspected outbreak was infected by different genotypes lacking resistance mutations. This shows that the selection of a genotype adapted to infect SOT recipients may have not occurred in this RTR.

The *P. jirovecii* genotype suspected in the present study to have caused an outbreak is distinct from five others involved in earlier outbreaks among SOT recipients. This suggests that different genotypes can adapt to infect SOT recipients independently and cause outbreaks, as previously reported (15). The genotype observed in Switzerland was potentially resistant to mycophenolate due to the presence of the resistance mutation in the IMPDH gene, like the genotype observed in France (16). In the Swiss-suspected outbreak, patient LA10 was ascertained to have received mycophenolate. Patients BE2 and BE3 probably also did as mycophenolate has been routinely prescribed in Bern since 1995, although we have not been able to obtain definite proof. This potential resistance has probably also driven the infections of the three RTRs, as reported in France (16). Consistently, the genotype involved in the earlier outbreak in Lyon among RTRs not receiving mycophenolate did not harbor the IMPDH resistance mutation. On the other hand, in this latter outbreak, the DHPS mutation M2 probably enabled to escape from suboptimal PCP prophylaxis using sulfadoxine (17).

The genotype involved in the outbreak suspected in the present study displayed a stable *msg*-I genomic repertoire over 8 years. Such stability has been previously observed over 5 years in six outbreaks among SOT recipients using restriction fragment length polymorphism of the second half of the *msg*-I genes (7, 8). This stability contrasts with the diversity of repertoires seen in non-outbreak patients in our previous study (22). This diversity is likely due to homologous recombinations leading to *msg*-I gene translocations within the subtelomeres and gene mosaicism (22). A low recombination frequency rate specifically within SOT recipients might explain the long-term stability of the genomic repertoires. Given the presence of a single genotype, this may also be due to a lack of mating events blending subtelomeres between co-infecting genotypes. Alternatively, the recombination frequency rate might always be low during the propagation of *P. jirovecii* microorganisms. In this latter scenario, the large diversity of repertoires present in non-outbreak patients might be produced within a huge population of colonized individuals harboring many generations of the fungus.

We observed a variation of the *msg*-I alleles expressed from almost the same genomic repertoire in the three RTRs of the suspected outbreak, with different subsets expressed. The expression of all alleles of the genomic repertoire in patient BE2 might be related to low immunosuppression and/or a long-lasting infection allowing multiplication of the subpopulations of microorganisms expressing all antigens. Although expected for a family of genes, this is the first time that selective expression of genes encoding antigens from the same repertoire is reported in *Pneumocystis*. This phenomenon might be crucial for the pathogenesis of PCP, as it may allow *P. jirovecii* to evade the immune response specific to each patient by varying surface antigens. Such antigenic variation likely arises from genetic recombinations at the conserved recombination junction element present at the beginning of each *msg*-I gene. These recombinations would generate over time new subpopulations of microorganisms expressing different *msg*-I alleles, which are then driven by the immune selective pressure. This scenario would be compatible with the exchange of the *msg*-I expressed allele over time proposed in our model for the antigenic variation system of *P. jirovecii* (20, 22). However, the selective expression that we report here would reduce the diversity of antigens expressed in each patient. This, in turn, would reduce the probability of escaping from the new immune system encountered upon a new interhuman transmission and thus to propagate. A plausible hypothesis is that all *msg*-I alleles of the repertoire are expressed in each *P. jirovecii* population, with a portion of them at low levels that are undetectable using our generic PCRs. This would allow subpopulations of *P. jirovecii* expressing various antigens to persist without being eliminated. This latter hypothesis would be consistent with the expression of all *msg*-I genes observed in the *Pneumocystis* species infecting specifically rats and mice, also in very varying abundance (19). Expression of all antigens in a low abundant population might be tolerated by a valid immune system because it may mimic the pulmonary flora that is present in this non-sterile niche (27). Nevertheless, this latter hypothesis might be invalidated if the alveoli where *Pneumocystis* microorganisms dwell are proven to be sterile, which presently cannot be excluded.

In conclusion, selective expression of *P. jirovecii* surface antigens might have played a role in the pathogenesis of PCP during the outbreak suspected in the present study by allowing escape from the immune response specific to each RTR patient. This phenomenon is reported here for the first time in *Pneumocystis* and constitutes a rare suggestion of the function of the antigenic variation systems harbored by *P. jirovecii* and other human pathogens, e.g., *Plasmodium* and *Trypanosoma* (28, 29). The potential resistance to mycophenolate of the *P. jirovecii* genotype has probably also driven the infections during the suspected outbreak. Besides, our observations support the concept that various *P. jirovecii* genotypes can adapt to infect SOT recipients, facilitating outbreaks.

## MATERIALS AND METHODS

### Samples and DNA extraction

Broncho-alveolar lavage (BAL) samples were collected from immunocompromised patients with PCP in two Swiss cities, i.e., Lausanne and Bern (Table S1). The patients were enrolled randomly. The patient inclusion criteria were (i) to be positive for *P. jirovecii* by real-time PCR and (ii) to have leftover BAL available for research. Unfortunately, the quantities of samples BE6 to BE9 available were not sufficient to determine the *P. jirovecii msg*-I repertoires that they contained. DNA was extracted from 0.2 mL of each BAL using the QIAamp DNA Blood Mini Kit (no. 51104, Qiagen). The three DNA samples from Lyon, France, were frozen at −80°C since our previous study (17).

### Characterization of the repertoires of *P. jirovecii msg*-I alleles

The methodology for characterizing the genomic and expressed repertoires of *P. jirovecii msg*-I alleles was described previously (22). Briefly, the repertoires were amplified from *P. jirovecii* DNA by generic PCRs using a forward primer located either within the conserved recombination junction element present at the beginning of each *msg*-I gene or within the promoter specific to *msg*-I genes present at a single copy per genome. For both PCRs, the reverse primer was located within a conserved region located at 90 bps after the stop codon of each *msg*-I gene. The PCR products were sequenced using PacBio CCS with long reads allowing analysis of the highly repetitive *msg* genes. A dedicated bioinformatics pipeline was developed to identify and quantify the *P. jirovecii msg*-I alleles present in each PCR product. Briefly, the PacBio CCS raw reads were cleaned, filtered, and clustered into “alleles” with a sequence identity threshold of 99.5%. This threshold corresponds to the error rate of the CCS method.

### Absence of laboratory mix-up

The legitimate assumption of a laboratory mix-up that resulted in almost identical genomic repertoires in the three RTRs of the suspected outbreak was addressed by checking the course of our experiments. Any cross contamination was excluded by the fact that these samples were processed at several steps of the procedure together with samples of patients harboring different repertoires (BE1, BE4, BE5, LA1, LA2, LA3, LA5, and LA8). Moreover, two control plasmids carrying a single allele were not contaminated by samples harboring many different alleles in our previous study (22).

#### Genotyping using multilocus sequence typing

Genotyping of *P. jirovecii* was performed as previously described by sequencing plasmid subclones of each PCR product (20). Direct sequencing of PCR products was also used as described in Table 1. The primers used were previously described: internal transcribed spacer number 1 of the nuclear rRNA genes operon (30), intron of the nuclear 26S rRNA gene (30), the variable region of the mitochondrial 26S rRNA gene (31), the region surrounding intron number 6 of the β-tubulin gene (30), internal half of cytochrome b (32), the region spanning the mutation site in dihydropteroate synthase (24; the A_HUM_ primer has been shortened: 5′-GCGCCTACACATATTATGGC-3′), and the region spanning the mutation site in inosine 5′-monophosphate dehydrogenase (16).

The alleles’ names and numbers shown are according to reference 30, except for CytB that are from reference 32. Allele 26S no. 6 and no. 9 have been described in reference 33 and allele 26S no. 4 in reference 22. The new ITS1 alleles B7 and B8 were named following the alleles’ names of reference (24). The new ITS1 allele B7 is identical to allele B except in positions 17–18 with four instead of two Ts, position 22 with C instead of T, and positions 85–91 with five instead of six Ts. The new ITS1 allele B8 is identical to allele B except in position 11 with G instead of A. The new mt26S allele no. 15 is named following the alleles’ names of reference 30 and is identical to allele no. 3 except in position 85 with A instead of C.

Part of the genotyping of the patients involved in the “Lyon” outbreak was published in reference 15 and confirmed in the present study. The alleles of the “Danish” and “Swiss-German” outbreaks were previously published in references 8, 15. Part of the genotyping of patients LA2, LA3, LA7, LA8, LA9, and B2 was previously published in the supplementary Table 7 of reference 22.

## Data Availability

The PacBio circular consensus sequencing raw reads analyzed in the present study have been deposited in the NCBI Sequence Read Archive in the frame of our previous study (22) and are accessible through accession code SRP434110. The msg-I raw data are accessible through accession numbers SRR24284242–SRR24284301 in BioProject accession number PRJNA936793. The sequences have been deposited in Genbank (1007 msg-I alleles) under accession numbers OR489167–OR490173. Only two new msg-I alleles (BE2ua208340226 and BE2ub147128807) were identified in the present study by the new analyses of the expressed repertoire of patient B2. They have been deposited in Genbank under accession numbers PP995154 and PP995155. The raw data of the two new analyses of the expressed repertoires of patients BE2, BE3, and LA10 are accessible through accession numbers SRR29713039–SRR29713044 in BioProject accession number PRJNA936793 of our previous study (22). The relative abundance of each msg-I allele identified in each patient is provided in Supplemental Material and Table S13. Computer codes used to generate results are deposited at https://doi.org/10.5281/zenodo.8363954. They were developed during our previous study (22). All data are available in the main text or the Supplementary Materials. All materials are available from the corresponding author.

## SUPPLEMENTAL MATERIAL

Supplemental Material mbio.00692-25-s0001.docx Supplemental information, Fig. S1, and Table S1.

Table S2 mbio.00692-25-s0002.xlsx Abundance of the *msg-I* alleles in the repertoires present in the 15 patients.

Table S3 mbio.00692-25-s0003.xlsx Abundance of the *msg-I* alleles in the triplicate analyses of the expressed repertoires of some patients involved in the outbreak.

ASM does not own the copyrights to Supplemental Material that may be linked to, or accessed through, an article. The authors have granted ASM a non-exclusive, world-wide license to publish the Supplemental Material files. Please contact the corresponding author directly for reuse.
