# Supplementary material for: Selective expression of Pneumocystis antigens in different patients during a suspected outbreak of Pneumocystis pneumonia
Source: mBio. 2025 Apr 17;16(5):e00692-25. doi: 10.1128/mbio.00692-25 (PMC12077189; doi:10.1128/mbio.00692-25)
Supplement: Supplemental Material — Supplemental information, Fig. S1, and Table S1. [file mbio.00692-25-s0001.docx]

Revised version cleaned.

**Supplemental Material**

**Supplementary Information.**

Limitation of the determination of the repertoires of *msg*-I alleles………….....………..2

**Figure S1.**

Composition of the expressed *msg*-I repertoires present in three renal transplant recipients with PCP involved in the suspected outbreak… ………………………..……3

**Table S1.**

Characteristics of the immunocompromised patients analysed in this study………….4

**Supplementary Information.** Limitation of the determination of the repertoires of *msg*-I alleles.

Duplicate analyses showed that the major limitation of the methodology is a varying efficiency of amplification and/or PacBio sequencing of the different alleles for unknown reasons, some being not detected at all (22). This results in an underestimation of the number of alleles per repertoire to an unknown extent. This also leads to a poor reproducibility of detection of the alleles that are low abundant in the PCR product. For example, the single allele present in the genomic repertoire of patient BE3 but absent in patient BE2 may have been missed in patient BE2 because it was low abundant (line 106 of Table S2). Similarly, the six alleles present in the expressed repertoire of patient BE2 but absent in the genomic repertoires of patients BE2, BE3, and LA10 of the suspected outbreak were also in low abundance (lines 86-91 of Table S2). Furthermore, the two and three additional alleles present in the repertoires of patients BE2 and BE3 relatively to that of LA10 were in low abundance, i.e., <2% of all reads composing the repertoire (Table S2). This suggests that they may have been missed in the repertoire of LA10 because of the methodological limitation. However, real modifications of the repertoire cannot be excluded, especially because of the long period of time of seven years between the PCPs.

Besides, the composition of the expressed repertoires is less reproducible and their numbers of alleles more underestimated than those of the genomic ones. This presumably results from the important variation of allele abundances corresponding to those of the subpopulations generated by the mutually exclusive expression of the *msg*-I family.

This methodological limitation explains probably the following feature of the results. The design of the generic PCRs used implies that each expressed repertoire should be a subset of its corresponding genomic repertoire. This was the case for 13 of the 15 patients investigated in the present study. For patients BE2 and LA2, the genomic repertoire missed respectively seven and five expressed alleles. All these 12 alleles represented <2% of the reads of the repertoire (Table S2), and thus may have not been detected by the amplification of the genomic repertoire. Consistently, such cases were also a vast minority in our previous study [three cases out of 24, Ref. (22)].

**Figure S1.** Composition of the expressed *msg*-I repertoires present in three renal transplant recipients with PCP involved in the suspected outbreak.

The expressed repertoires present in the three patients involved in the suspected outbreak were analysed in triplicate (the analysis shown in Fig. 2, plus analyses a and b shown in Fig. S1). Each vertical line of the heatmap represents an allele present in the given repertoire with the color representing its abundance in % of all reads composing the repertoire, as indicated at the top left of the figure. The number of alleles present in the patient are indicated next to the patient’s code. The 66 distinct alleles identified in the repertoires were sorted using a hierarchical classification tree of the multiple alignment of the allele sequences (Fitch distance, average linkage). The patients were sorted using a tree of presence/absence of each allele in their repertoire (binary distance, average linkage). LA, Lausanne. BE, Bern. Data are provided in Table S3.


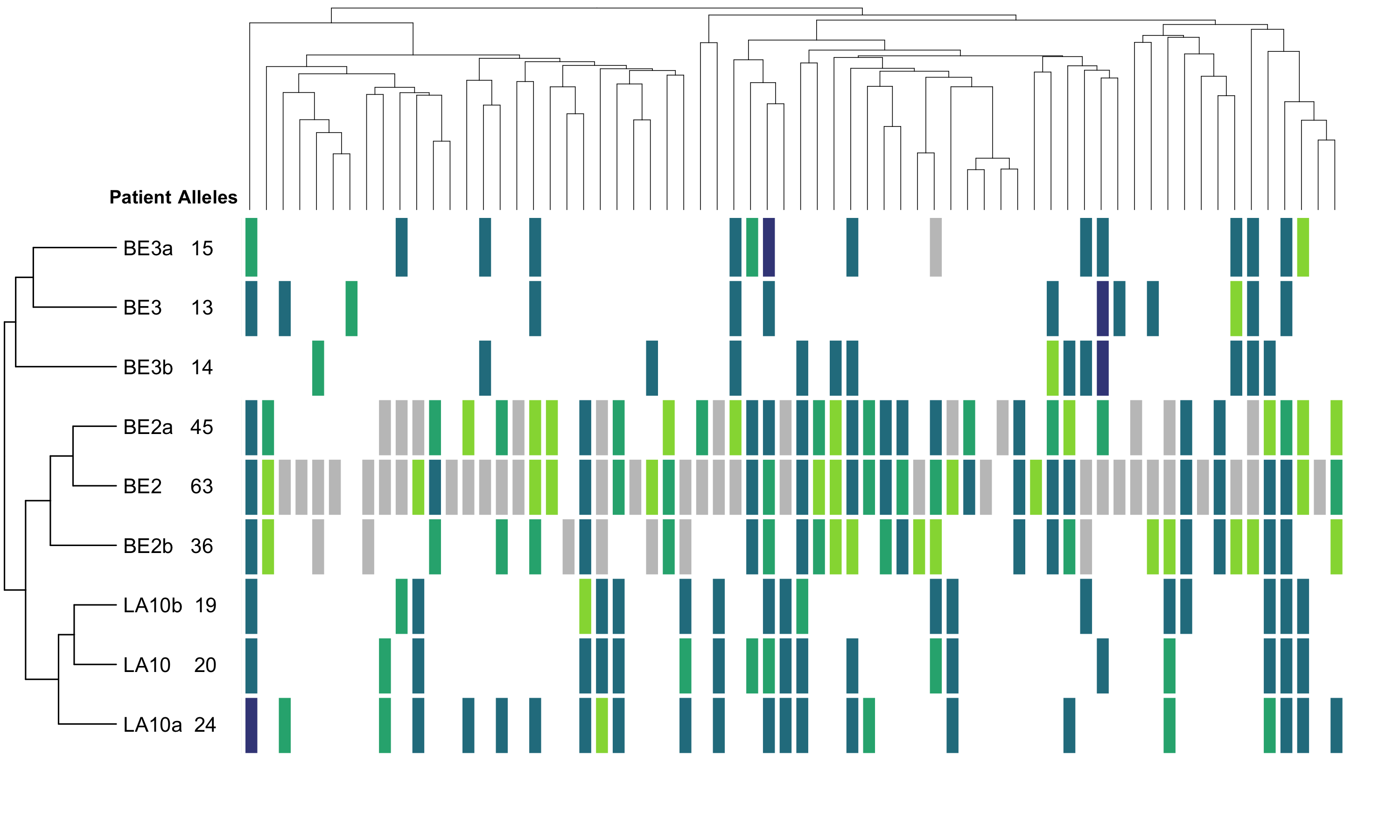

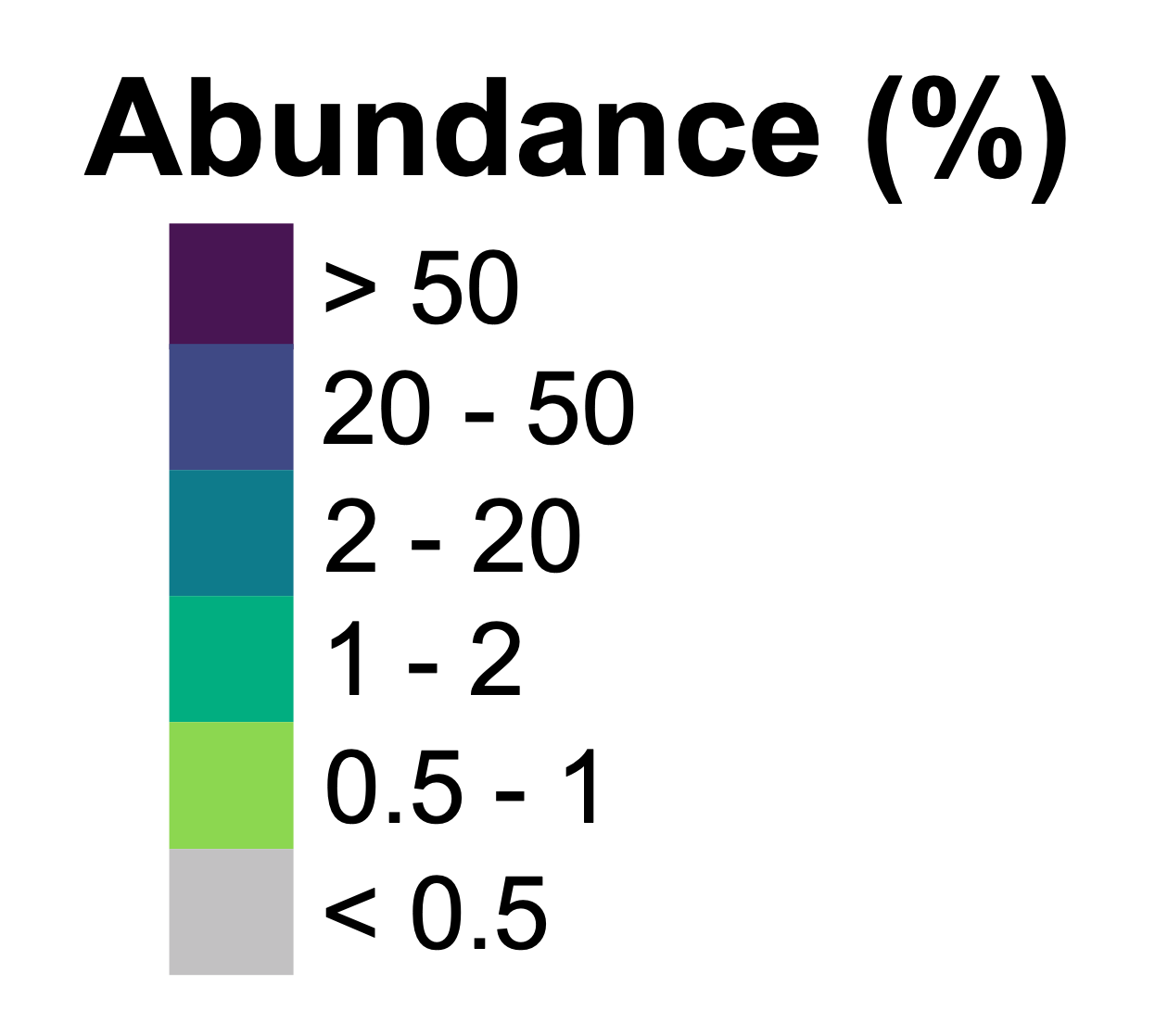


66 alleles

Table S1. Characteristics of the 15 immunocompromised patients analysed in this study.

| **City** | **Country** | **Patient code** | **Collection year** | **Underlying disease** |
| --- | --- | --- | --- | --- |
| Lausanne | Switzerland | LA1 | 2014 | HIV |
|  |  | LA2 | 2014 | HIV |
|  |  | LA3 | 2014 | unknown |
|  |  | LA4 | 2018 | unknown |
|  |  | LA5 | 2014 | unknown |
|  |  | LA6 | 2017 | unknown |
|  |  | LA7 | 2014 | HIV |
|  |  | LA8 | 2012 | HIV |
|  |  | LA9 | 2014 | HIV |
|  |  | LA10 | 2021 | kidney transplant |
| Bern | Switzerland | BE1 | 2014 | HIV |
|  |  | BE2 | 2013 | kidney transplant |
|  |  | BE3 | 2014 | kidney transplant |
|  |  | BE4 | 2015 | cancer |
|  |  | BE5 | 2014 | cancer |
